# The addition of bevacizumab in the first-line treatment for metastatic colorectal cancer: an updated meta-analysis of randomized trials

**DOI:** 10.18632/oncotarget.20314

**Published:** 2017-08-17

**Authors:** Hyun Joo Jang, Bum Jun Kim, Jung Han Kim, Hyeong Su Kim

**Affiliations:** ^1^ Division of Gastroenterology, Department of Internal Medicine, Dongtan Sacred-Heart Hospital, Hallym University Medical Center, Hallym University College of Medicine, Hwasung 18450, Republic of Korea; ^2^ Division of Hemato-Oncology, Department of Internal Medicine, Kangnam Sacred-Heart Hospital, Hallym University Medical Center, Hallym University College of Medicine, Seoul 07441, Republic of Korea; ^3^ Department of Internal Medicine, Korean Armed Forces Capital Hospital, The Armed Forces Medical Command, Sungnam 13574, Republic of Korea

**Keywords:** colorectal cancer, bevacizumab, irinotecan, meta-analysis

## Abstract

Bevacizumab has shown survival benefits when added to chemotherapy in patients with metastatic colon cancer (mCRC). However, the efficacy of bevacizumab may depend on the accompanying chemotherapeutic regimen. We performed this meta-analysis to examine the impact of the choice of chemotherapy regimen on the survival benefits of bevacizumab in the first-line treatment for patients with mCRC. Electric databases were searched for eligible randomized trials. From 9 studies, 3,710 patients with mCRC were included in the meta-analysis of hazard ratios (HRs) for progression-free survival (PFS) or overall survival (OS). Compared with chemotherapy alone, the addition of bevacizumab to chemotherapy significantly prolonged PFS (HR = 0.66 [95% confidence interval (CI), 0.55–0.77], *P* < 0.0001) and OS (HR = 0.84 [95% CI, 0.77–0.92], *P* = 0.0001). In the subgroup analysis according to the chemotherapeutic regimens, bevacizumab showed both PFS (HR = 0.57 [95% CI, 0.41–0.77], *P* = 0.0004) and OS (HR = 0.79 [95% CI, 0.67–0.93], *P* = 0.004) advantages only in combination with irinotecan-based regimen. In conclusion, this meta-analysis confirms that the addition of bevacizumab to chemotherapy significantly prolongs PFS and OS in the first-line treatment for mCRC. The subgroup analyses suggest that irinotecan-based regimen may be a better partner of bevacizumab in terms of both PFS and OS.

## INTRODUCTION

Colorectal (CRC) is the third most common cancer and the second leading cause of cancer-related death worldwide [1, 2]. Approximately 80% of patients with CRC have resectable disease at the time of diagnosis [3], but 30–50% of patients who undergo curative surgery experience disease recurrence and die of metastatic diseases [4]. Standard treatment for non-resectable metastatic CRC (mCRC) is combination chemotherapy with or without a targeted molecular agent.

For more than four decades, 5-fluorouracil (5-FU) plus leucovirin (LV) was the only approved regimen to treat mCRC, producing mild survival benefit over best supportive care [5–6]. In 1990s, the introduction of irinotecan or oxaliplatin into the 5-FU-based regimen led to a significant increase in overall response rate (ORR), progression-free survival (PFS), or overall survival (OS) [7–8]. Capecitabine, an oral fluoropyrimidine, has been proved as an effective alternative to 5-FU in mCRC [9]. Since 2000, the addition of targeted agents such as bevacizumab, cetuximab, or panitumumab to standard chemotherapy has broadened treatment options with an additional survival benefits [10–19]. In clinical practice, various chemotherapy regimens [bolus 5-FU/LV (FL), capecitabine monotherapy, irinotecan plus bolus 5-FU/LV (IFL), irinotecan plus infusional 5-FU/LV (FOLFIRI), oxaliplatin plus infusional 5-FU/LV (FOLFOX), capecitabine plus oxaliplatin (XELOX)] can be used in combination with a targeted agent.

Cetuximab, a monoclonal antibody against epidermal growth factor receptor, has demonstrated its survival benefit in the first-line treatment for patients with *KRAS* wild-type mCRC [10, 11]. However, a recent meta-analysis of four randomized trials found that only patients treated with infusional 5-FU-based chemotherapy, not those with capecitabine/bolus 5-FU-based chemotherapy, derived benefit from cetuximab [20]. This finding suggests that the efficacy of cetuximab may depend on the choice and schedule of fluoropyrimidine.

Bevacizuamb is a humanized recombinant monoclonal antibody that blocks all isoforms of vascular endothelial growth factor-A. Several randomized clinical trials in patients with mCRC have demonstrated that bevacizumab in combination with chemotherapy improves PFS or OS [13–19]. However, the impact of the choice of accompanying chemotherapeutic regimen on the benefit of bevacizumab has not been revealed. In a randomized phase III trial with a 2 × 2 factorial design, statistical PFS superiority of bevacizumab versus placebo was evident in the XELOX subgroup [hazard ration (HR) = 0.77, *P* = 0.0026], but did not reach the significance level in the infusional 5-FU/LV plus oxaliplatin (FOLFOX-4) subgroup (HR = 0.89, *P* = 0.1871) [13]. Thus, the efficacy of bevacizumab might depend on the cytotoxic drugs combined. We performed this meta-analysis of randomized controlled trials to examine the impact of the choice of chemotherapy regimen on the effect of bevacizumab in the first-line treatment for patients with mCRC.

## RESULTS

### Results of search

**Figure 1:**
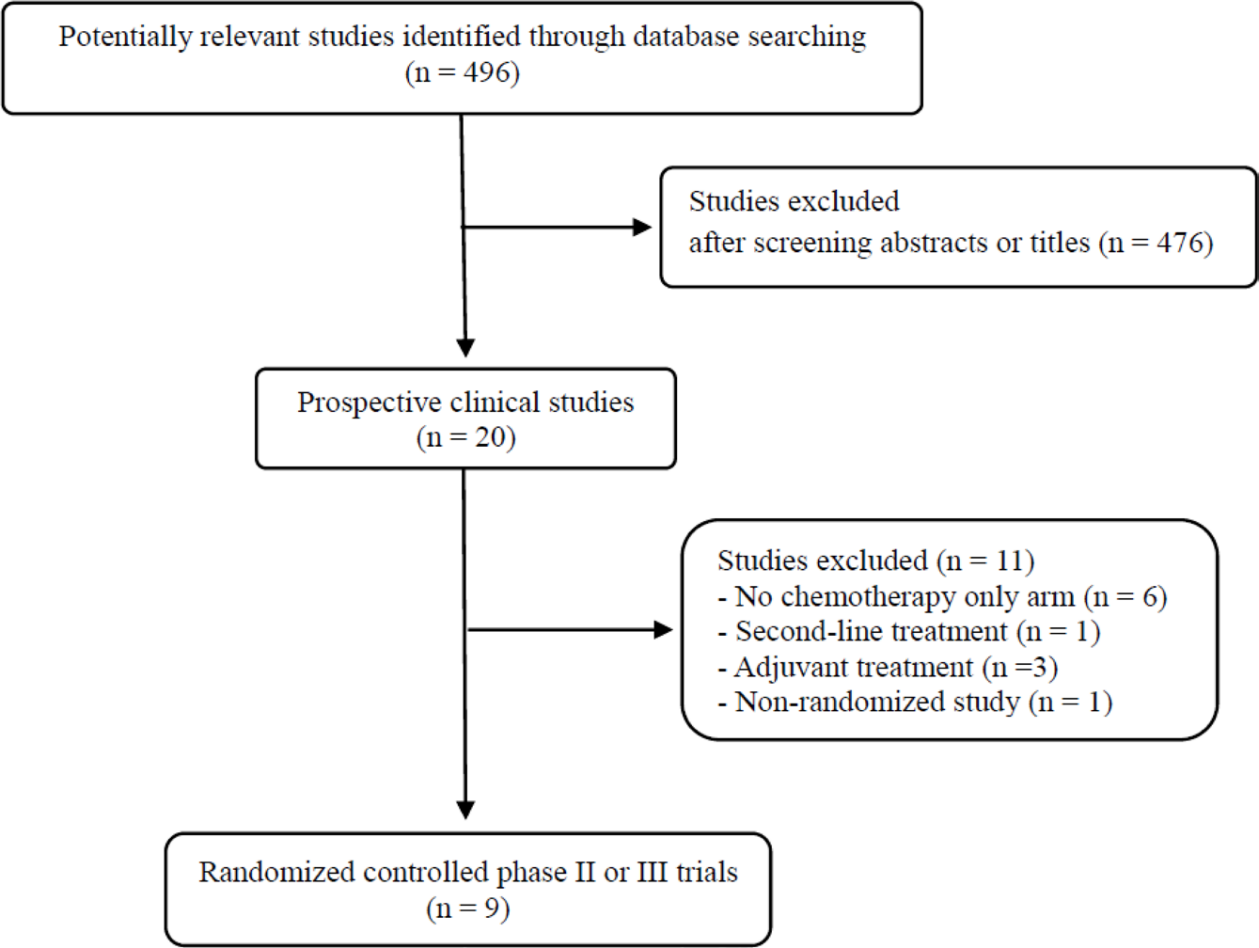
Flow diagram of search process

Figure 1 shows the search process based on the applied keywords and inclusion criteria. A total of 496 potentially relevant studies were identified and screened by the search strategy; 476 were excluded after screening the titles and abstracts. Of the remaining 20 prospective studies, eleven were further excluded by the inclusion criteria: six prospective trials with no chemotherapy only control arm, three evaluating chemotherapy plus bevacizumab as adjuvant treatment, one conducted in second-line setting, and the remaining one non-randomized phase II trial. Finally, nine randomized phase II or III trials were included in this meta-analysis [13–19, 21, 22].

### Characteristics of the eligible studies

Table 1 shows the main characteristics and outcomes of the included randomized trials. We also summarized treatment regimens used in each arm. Seven phase III trials [14, 16–19, 21, 22] and two phase II trials [13, 15] were included. In most studies except for three [15, 16, 21], the primary endpoint was PFS.

**Table 1:** Summary of the nine included studies

Author, (year)	Phase	No. of patients	Treatment arms	Primary endpoint	ORR	mPFS (mo)	HR for PFS (95% CI)	mOS (mo)	HR for OS (95% CI)
Kabbinavar *et al*., (2003)	II	36	Bolus 5-FU 500 mg/m^2^ + LV 500 mg/m^2^ wkly for the first 6 wks of each 8-wk cycle.	ORR PFS	17%	5.2	0.54 (0.33–0.88) (FL ± Bev) *P* = 0.003	13.8	na
		35	Same with bevacizumab 5 mg/kg every 2 wk		40%	9.0		21.5	
		33	Same with bevacizumab 10 mg/kg every 2 wk		24%	7.2		16.1	
Hurwitz *et al*., (2004)	III	411	Irinotecan 125 mg/m^2^ + bolus 5-FU 500 mg/m^2^+ LV 20 mg/m^2^ once wkly for 4 wk every 6 wk	OS	34.8%	6.2	0.54 (0.37–0.78) *P* < 0.001	15.6	0.66 (0.52–0.85) *P* < 0.001
		402	Same with bevacizumab 5 mg/kg every 2 wk		44.8%	10.6		20.3	
Kabbinavar *et al*., (2005)	II	105	Bolus 5-FU 500 mg/m^2^ + LV 500 mg/m^2^ wkly for the first 6 wk of each 8-wk cycle.	OS	15.2%	5.5	0.50 (0.34–0.74) *P* = 0.0002	12.9	0.78 (0.56–1.10) *P* = 0.16
		104	Same with bevacizumab 5 mg/kg every 2 wk		26.0%	9.2		16.6	
Stathopoulos *et al*., (2010)	III	108	Bolus 5-FU 500 mg/m^2^ + LV 200 mg/m^2^ with irinotecan 135 mg/m^2^ every 3 wk	OS	35.2%	na	na	25	1.05 (0.81–1.36) *P* = 0.139
		114	Same with bevacizumab 7.5 mg/kg every 3 wk		36.8%	na		22	
Saltz *et al*., (2008)	III	701 (350/351)	XELOX (capecitabine 1000 mg/m^2^twice daily on days 1–14 + oxaliplatin 130 mg/m^2^ on day 1 every 3 wk) or FOLFOX-4 (oxaliplatin 85 mg/m^2^ on day 1 with LV 200 mg/m^2^ followed by 5-FU bolus 400 mg/m^2^ and 600 mg/m^2^ 22-h iv infusion for 2 days every 2 wk)	PFS	47%	8.0	0.83 (0.74–0.93) (Ctx ± Bev) *P* = 0.0023 *XELOX ± Bev: 0.77 (0.65–0.92) *P* = 0.0026 *FOLFOX4 ± Bev: 0.89 (0.74–1.06) *P* = 0.1871	19.9	0.89 (0.79–1.00) (Ctx ± Bev) *P* = 0.077 *XELOX ± Bev: 0.84 (0.71–1.01) *FOLFOX-4 ± Bev: 0.94 (0.77–1.15)
		699 (350/349)	XELOX + bevacizumab 7.5 mg/kg every 3 wk or FOLFOX-4 + bevacizuamb 5 mg/kg every 2 wk		49%	9.4		21.3	
Tebutt *et al*., (2010)	III	156	Capecitabine 1000 or 1250 mg/m^2^ twice daily on days 1–14 every 3 wk	PFS	30.3%	5.7	0.61 (0.50–0.74) (C/CM ± Bev)	18.9	na
		157	Capecitabine (same) + bevacizumab 7.5 mg/kg every 3 wk		38.1%	8.5	0.62 (0.49–0.79) (C ± Bev) *P* < 0.001	18.9	0.88 (0.68–1.13) (C ± Bev) *P* = 0.314
		158	Capecitabine (same) + mitomycin 7 mg/m^2^ every 6 wks+ bevacizumab 7.5 mg/kg every 3 wk		45.9%	8.4	0.56 (0.44–0.71) (C vs CM + Bev) *P* < 0.001	16.4	0.94 (0.73–1.21) (C vs CM + Bev) *P* = 0.642
Cunningham *et al*., (2013)	III	140	Capecitabine 1000 mg/m^2^twice daily on days 1–14, every 3 wk	PFS	10%	5.1	0.53 (0.41–0.69) *P* < 0.0001	16.8	0.79 (0.57–1.09) *P* = 0.18
		140	Same withbevacizumab7.5 mg/kg on day 1 every 3 wk		19%	9.1		20.7	
Guan *et al*., (2011)	III	79	Irinotecan 125 mg/m^2^ + bolus LV 20 mg/m^2^and 5-FU 500 mg/m^2^ iv infusion over 6–8 h wkly for 4 wk every 6 wk	PFS	17%	4.2	0.44 (0.31–0.63) *P* < 0.001	13.4	0.62 (0.41–0.95) *P* = 0.014
		142	Same with bevacizumab 5 mg/kg every 2 wk		35%	8.3		18.7	
Passardi *et al*., (2015)	III	194 (76/118)	FOLFIRI (irinotecan 180 mg/m^2^ on day 1 with 5-FU 400 mg/m^2^ bolus and 600 mg/m^2^ by 22-h infusion + LV 200 mg/m^2^ on days 1 and 2 every 2 wk) or FOLFOX-4	PFS	50%	8.4	0.86 (0.70–1.07) *P* = 0.182 *FOLFIRI ± Bev: 0.75 (0.54–1.05) *FOLFOX4 ± Bev: 1.00 (0.76–1.33)	21.3	1.13 (0.89–1.43) *P* = 0.317 *FOLFIRI ± Bev: na *FOLFOX4 ± Bev: na
		176 (73/103)	FOLFIRI or FOLFOX-4 with bevacizumab 5 mg/kg every 2 wk		50.6%	9.6		20.8	

Bev, bevacizumab; C, capecitabine; M, mitomycin; Ctx, chemotherapy; 5-FU, 5-fluorouracil; LV, leucovorin; FL, 5-fluorouracil plus leucovorin; ORR, overall response rate; CI, confidence interval; mOS, median overall survival; mPFS, median progression-free survival; HR, hazard ratio; wk, week; na, not available.

### Progression-free survival

**Figure 2:**
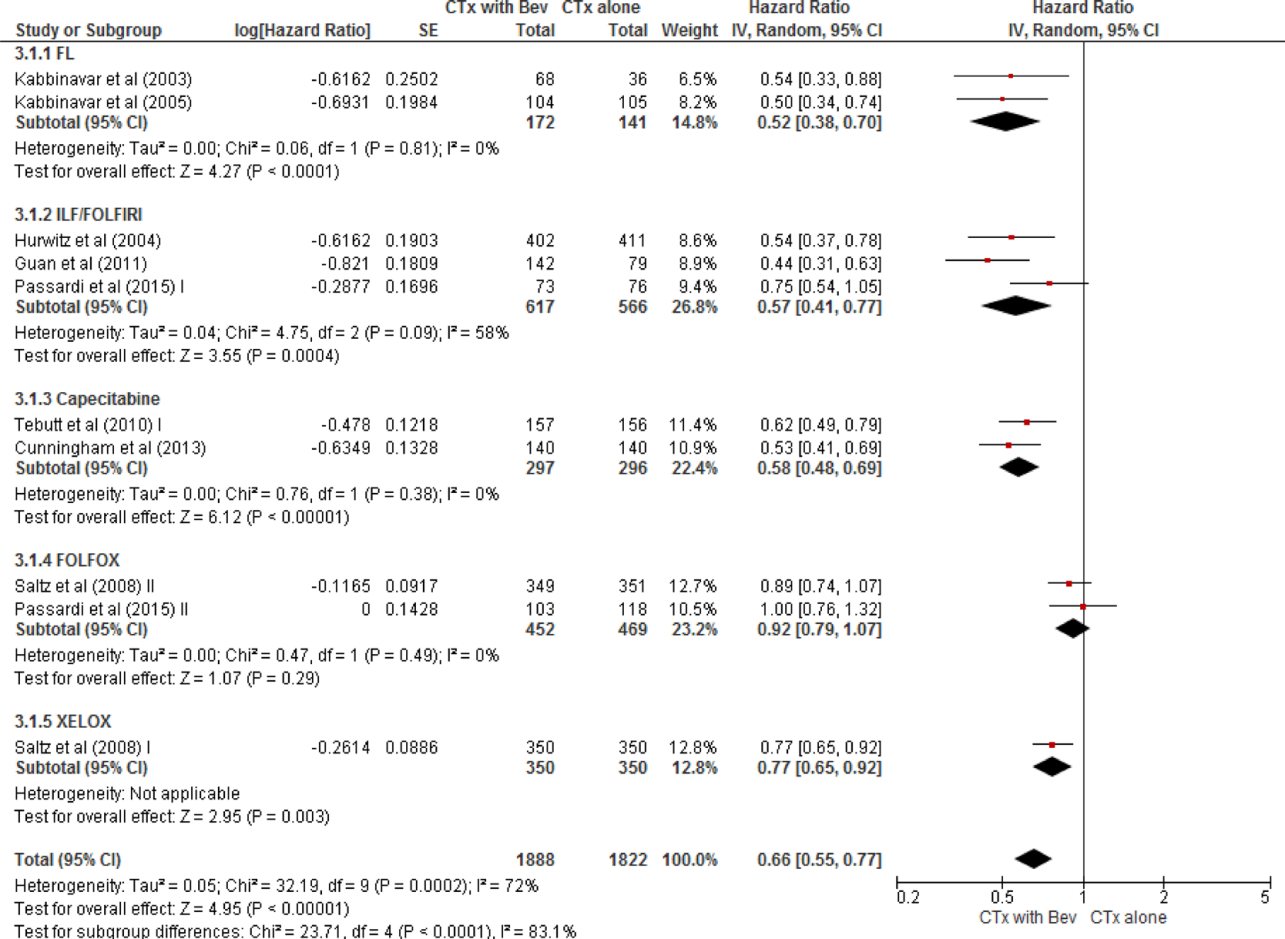
Forest plot for progression-free survival

From the nine studies [13–19, 21, 22], a total of 3,710 patients (1,822 in chemotherapy alone group and 1,888 in bevacizumab plus chemotherapy group) were included in the meta-analysis of HRs for PFS. Compared with chemotherapy alone, bevacizumab combined with chemotherapy significantly prolonged PFS (HR = 0.66 [95% confidence interval (CI), 0.55–0.77], *P* < 0.0001) (Figure 2). We adopted the random-effects model because there was a significant heterogeneity among studies (*X*^2^ = 32.19, *P* = 0.0002, *I^2^* = 72%).

In the subgroup analysis according to the chemotherapy regimens in combination with bevacizumab, FL (HR = 0.52 [95% CI, 0.38–0.70], *P* < 0.0001), IFL/FOLFIRI (HR = 0.57 [95% CI, 0.41–0.77], *P* = 0.0004), capecitabine (HR = 0.58 [95% CI, 0.48–0.69], *P* < 0.00001), and XELOX (HR = 0.77 [95% CI, 0.65–0.92], *P* = 0.003) were associated with a significant improvement of PFS. However, bevacizumab in combination with FOLFOX regimen failed to significantly prolong PFS, compared with FOLFOX alone (HR = 0.92 [95% CI, 0.79–1.07], *P* = 0.29).

### Overall survival

**Figure 3:**
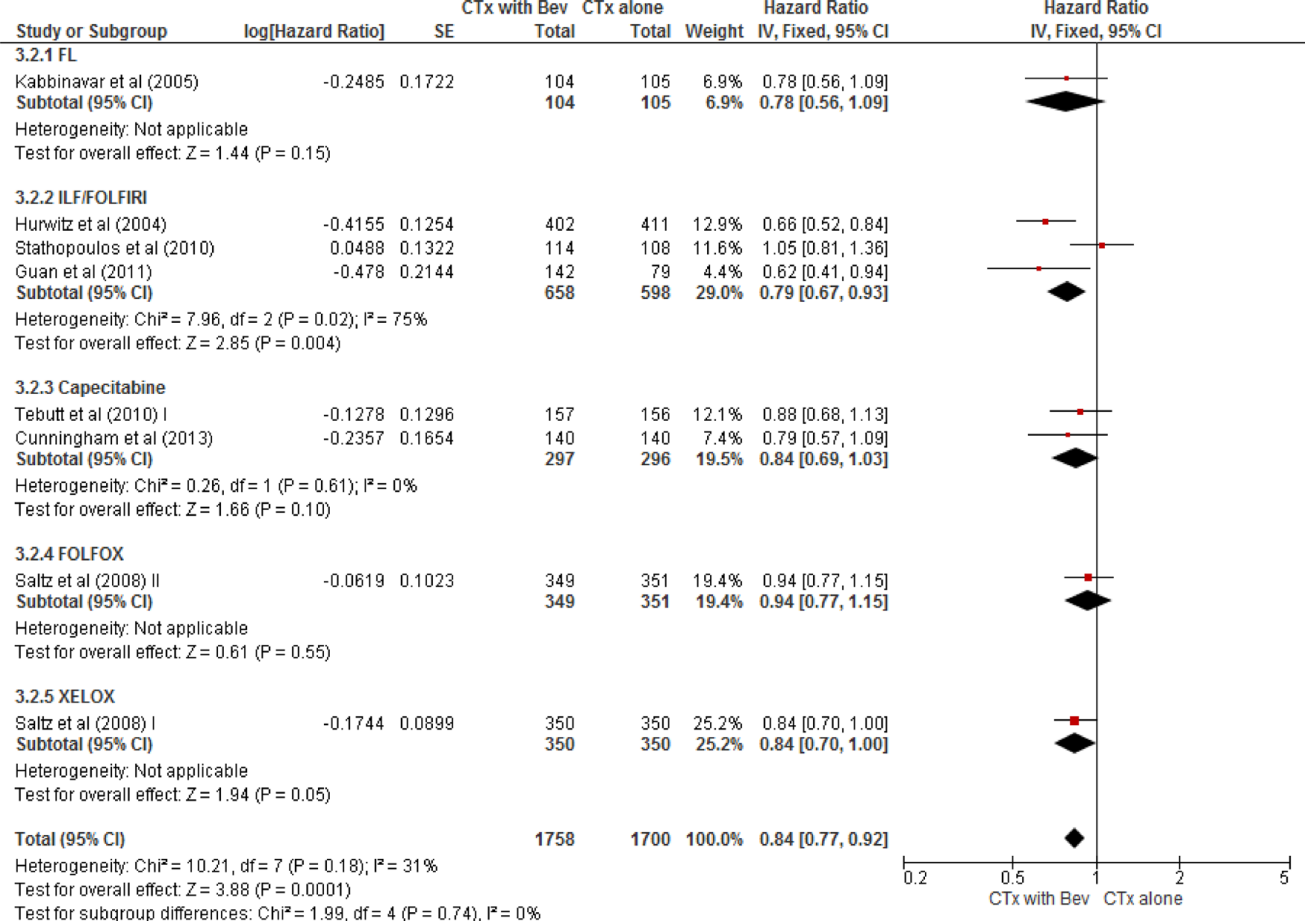
Forest plot for overall survival

From the nine studies [13–19, 21, 22], a total of 3,458 patients (1,700 in chemotherapy alone group and 1,758 in bevacizumab plus chemotherapy group) were included in the meta-analysis of HRs for OS. Compared with chemotherapy alone, the addition of bevacizumab to chemotherapy also significantly prolonged OS (HR = 0.84 [95% CI, 0.77–0.92], *P* = 0.0001) (Figure 3). We adopted the fixed-effects model because there was no significant heterogeneity among studies (*X*^2^ = 10.21, *P* = 0.18, *I^2^* = 31%).

In the subgroup analysis according to the chemotherapy regimens, FL (HR = 0.78 [95% CI 0.56–1.09], *P* = 0.15), capecitabine (HR = 0.84 [95% CI, 0.69–1.03], *P* = 0.10), and FOLFOX regimen (HR = 0.94 [95% CI, 0.77–1.15], *P* = 0.55) failed to significantly prolong OS in combination with bevacizumab. Compared with chemotherapy alone, however, IFL/FOLFIRI regimen combined with bevacizumab significantly prolonged OS (HR = 0.79 [95% CI, 0.67–0.93], *P* = 0.004) and XELOX plus bevacizumab tended to improve OS (HR = 0.84 [95% CI, 0.70–1.00], *P* = 0.05).

### Publication bias

**Figure 4:**
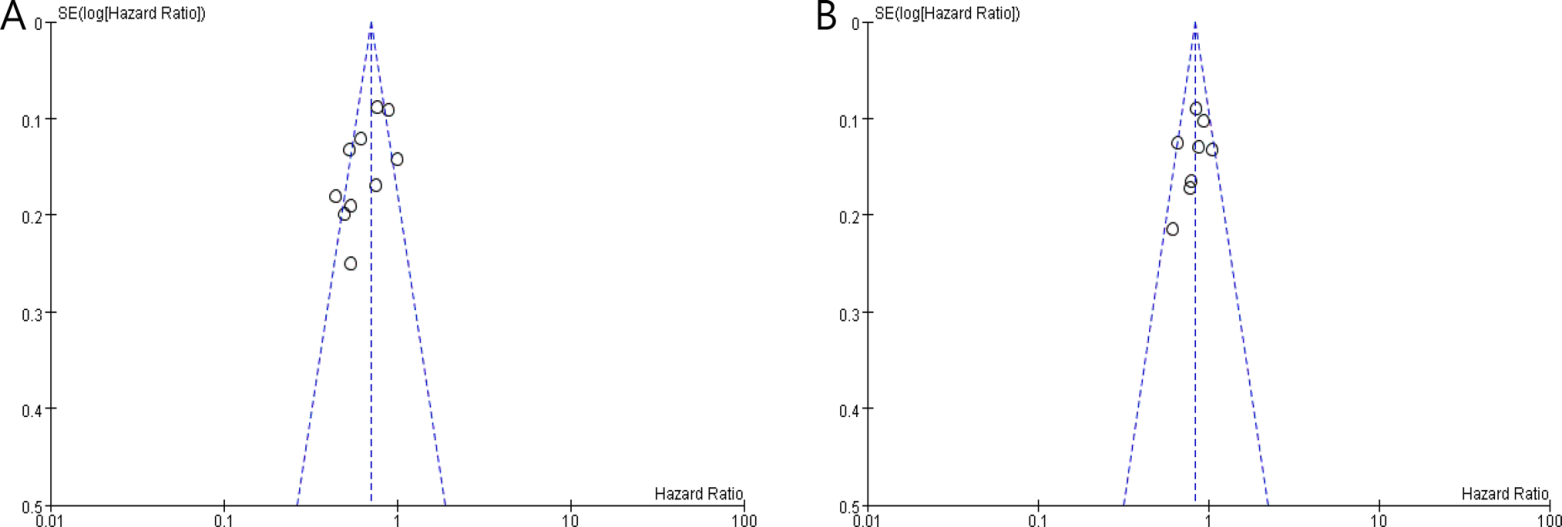
Funnel plots for publication bias test regarding progression-free survival (**A**) and overall survival (**B**).

The funnel plots are relatively symmetrical for both PFS and OS, indicating that the amount of publication bias in our meta-analysis is not substantial (Figure 4).

## DISCUSSION

Bevacizumab has been tested alongside various chemotherapy regimens in patient with mCRC [13–19, 21, 22]. While many studies have shown that the addition of bevacizumab to chemotherapy significantly prolongs PFS or OS [13–19], other studies have not confirmed their findings [22, 23]. The additional effect of bevacizumab on survival (PFS or OS) in patients with mCRC has been investigated in several meta-analyses [23–29]. Most studies indicated that the addition of bevacizumab to chemotherapy significantly prolonged both PFS and OS. In the subgroup analyses according to the involved chemotherapeutic agents, however, the survival benefits of bevacizumab were not in concordance among studies. These previous meta-analyses included a limited number of studies and adopted heterogeneous inclusion criteria (including non-randomized studies or studies with different treatment setting). Thus, we performed the current updated meta-analysis only with randomized controlled trials conducted in first-line treatment setting of mCRC. We also assessed the survival benefits of bevacizumab stratified by the choice of chemotherapeutic regimen, not according to the individual cytotoxic agents.

In our meta-analysis, chemotherapy combined with bevacizumab significantly prolonged PFS (HR = 0.66, *P* < 0.0001) compared with chemotherapy alone. The addition of bevacizumab to chemotherapy also significantly improved OS (HR = 0.84, *P* = 0.0001). Our results confirm the beneficial effects of adding bevacizumab to chemotherapy in terms of both PFS and OS. However, the survival benefits of bevacizumab were not consistent throughout the combined chemotherapeutic regimens. In the subgroup analysis, bevacizumab significantly improved both PFS (HR = 0.57, *P* = 0.0004) and OS (HR = 0.79, *P* = 0.004) only in combination with irinotecan-based regimens (ILF or FOLFIRI).

In a previous meta-analysis by Chen *et al*., the addition of bevacizumab to the usual chemotherapy regimens significantly improved PFS (HR = 0.68 [95% CI, 0.59–0.78], *P* < 0.00001), but not OS (HR = 8.89 [95% CI, 0.78–1.02], *P* = 0.08) [28]. In the subgroup analysis, the PFS benefit of bevacizumab was only observed when capecitabine-containing regimens were used. These findings may be due to insufficient data available at the time of meta-analysis. Recently Iliac *et al*. also published a meta-analysis in which a significant improvement was identified for both PFS (HR = 0.64 [95% CI, 0.55–0.73], *P* < 0.00001) and OS (HR = 0.84 [95% CI, 0.74–0.94], *P* = 0.003) when bevacizumab was combined with chemotherapy in patients with mCRC [29]. The subgroup analysis showed that the OS advantage was significant only for FL and oxalipatin-based regimens while the improvement of PFS remained throughout different chemotherapy regimens. However, this study included a non-randomized trial [30] and a randomized trial conducted in second-line setting [31].

Another meta-analysis by Macedo *et al*. found that bevacizumab significantly improved PFS (HR = 0.72, [95% CI, 0.66–0.78], *P* < 0.000001) and OS (HR = 0.84 [95% CI, 0.77–0.91], *P* < 0.00001) in combination with chemotherapy. This study included 6 randomized trials only conducted in first-line treatment setting of mCRC. Subgroup analyses supported the OS advantage with bevacizumab restricted to irinotecan-based regimens, which are consistent with our results. These findings suggest that irinotecan-based regimens might be a better partner of bevacizumab in terms of both PFS and OS. However, these meta-analyses have a limited number of trials in each subgroup, and OS can be affected by therapies following first-line treatment. Thus, further researches are needed to reveal the interaction of bevacizuamb and cytotoxic agents and identify the best chemotherapeutic regimen which can derive the most benefits in combination with bevacizumab.

Of note, our study has several limitations. First, the subgroup analyses stratified by chemotherapeutic regimens included a limited number of studies in each subgroup. Therefore, our findings need to be verified in further studies. Second, there was a significant heterogeneity observed among studies especially in the meta-analysis of PFS. We used the random-effects model to minimize its influence on the results. Finally, literature search only included studies written in English, which might lead to omitting studies published in another language.

In conclusion, this meta-analysis confirms that the addition of bevacizumab to chemotherapy significantly prolongs PFS and OS in the first-line treatment for patients with mCRC. The subgroup analyses suggest that irinotecan-based regimen (ILF or FOLFIRI) might be a better partner of bevacizumab in terms of both PFS and OS. Considering that a limited number of trials were included in this meta-analysis, however, further studies are warranted to explore the best chemotherapeutic combination with bevacizumab.

## MATERIALS AND METHODS

### Search strategy

A systematic review of the literature was carried out according to the predefined protocol [32]. Electric databases including PubMed, EMBASE, and Cochrane Library databases were searched up to the end of April 2017 for eligible articles. The following searching keywords were used: “vascular endothelial growth factor inhibitor or bevacizumab”, “colon cancer or colon neoplasm or colorectal cancer”, and “randomized.” All eligible studies were retrieved and their bibliographies were checked for other relevant publications. In case of duplicate publications, the most recent articles were selected.

### Study eligibility criteria

Eligible studies were required to meet the following inclusion criteria: (i) randomized trials conducted in patients with mCRC; (ii) randomization of patients in the first-line treatment setting to either chemotherapy alone or chemotherapy with becacizumab; (iii) providing HRs with 95% CIs for PFS or OS; (iv) papers written in English.

Case series, observational studies, and trials conducted in adjuvant or second-line setting were excluded.

### Data extraction

The following data were carefully extracted from all eligible studies: first author's name, year of publication, trial phase, the number of participants, treatment regimens, primary endpoints, overall response rates, median PFS and OS, and their HRs with 95% CIs.

Data extraction was done independently by two investigators (HSK and HJJ). If these two authors could not reach a consensus, other author (JHK) was consulted to resolve the disputes.

### Statistical analysis

Most statistical values used in this meta-analysis were collected directly from the original article. For papers with no HR or 95% CI, the Engauge Digitizer (version 9.1) was used to obtain the needed data from Kaplan-Meier curves. The effect size of PFS and OS was pooled through HR and its 95% CI. The heterogeneity across studies was examined by the *Q* statistic and the *I*^2^ statistic. The fixed-effects model (Mantel–Haenszel method) was selected for pooling the homogeneous outcomes when *P* ≥ 0.1 and *I*^2^ *≤* 50%, and the random-effects model (DerSimonian–Laird method) was applied for pooling heterogeneous outcomes when *P* < 0.1 and *I*^2^ > 50%.

All reported *P*-values were from two-sided versions of the respective test; *P* < 0.05 was considered statistically significant. Results were presented graphically as forest plots with diamonds representing estimate of the polled effect and the width of diamond representing its precision. The Review Manager software (version 5.2) was used to report outcomes. Publication bias was assessed graphically by the funnel plot method [33].
